# Real-world retrospective analysis of clinical outcomes in pediatric acquired aplastic anemia: a single-center 10-year cohort study

**DOI:** 10.3389/fped.2026.1915688

**Published:** 2026-08-13

**Authors:** Shanshan Li, Kai Chen, Hui Jiang, Na Zhang, Jingwei Yang, Xuelian Liao, Ting Zhang, Shayi Jiang, Jingbo Shao

**Affiliations:** 1 Department of Hematology and oncology, Shanghai Children’s Hospital, School of Medicine, Shanghai Jiao Tong University, Shanghai, China; 2 Department of Gastroenterology, Hepatology and Nutrition, Shanghai Children's Hospital, School of Medicine, Shanghai Jiao Tong University, Shanghai, China; 3 Gut Microbiota and Metabolic Research Center, Institute of Pediatric Infection, Immunity and Critical Care Medicine, School of Medicine, Shanghai Jiao Tong University, Shanghai, China

**Keywords:** acquired aplastic anemia, hematopoietic stem cell transplantation, immunosuppressive therapy, outcome, pediatric, thrombopoietin receptor agonist

## Abstract

**Introduction:**

Pediatric aplastic anemia (AA), a rare and potentially fatal disease, demonstrates significant heterogeneity in pathogenesis, disease severity, therapeutic regimens, and clinical outcomes.

**Methods:**

In this study, clinical features and outcomes of 70 children with AA, including severe AA (SAA, *n* = 30), very severe AA (vSAA, *n* = 21) and nonsevere AA (nSAA, *n* = 19), were retrospectively analyzed, with a median follow-up of 60.7 months (range, 0.4-136.5 months).

**Results:**

Patients with nSAA were mainly treated with cyclosporine A, whereas SAA/vSAA patients primarily received allogeneic hematopoietic stem cell transplantation (HSCT) followed by standard immunosuppressive therapy (IST). Ultimate therapy regimens differed significantly between patients with SAA/vSAA and nSAA (*p* = 0.001). SAA/vSAA group exhibited a higher overall response rate at the 2-year follow-up (85.5% vs. 55.6%, *p* = 0.019). The 2-year overall survival (OS) and event-free survival (EFS) for the entire cohort were 97.1% and 48.5%, respectively. In patients with SAA/vSAA, HSCT was associated with higher and faster cumulative complete response (CR) rate (*p* < 0.0001) and superior EFS (*p* = 0.0297) compared with IST. Two IST-resistant patients were observed to achieved CR with eltrombopag (EPAG) salvage therapy.

**Discussion:**

pediatric AA carries excellent OS but suboptimal EFS. HSCT tends to yield more favorable EFS compared with IST in SAA/vSAA patients, and EPAG may act as an effective salvage option for appropriately selected IST-resistant individuals. Further multicenter prospective research is warranted prior to implementing these findings in routine clinical practice.

## Introduction

Aplastic anemia (AA) is a rare condition with an estimated incidence rate of 2–2.3 cases/million per year (7.4 cases/million per year in Asia) and arises due to the destruction of hematopoietic cells by a variety of mechanisms (1, 2). The possible reasons for the higher prevalence of AA in East Asia involve multiple factors, including genetic background, environmental exposure and differences in medical diagnosis and registration systems (3). AA is a descriptive term that is used for a combination of hypoplastic or aplastic bone marrow disorders and a variable degree of paucity in at least two of the three main cell lines: red cells, white cells and/or platelets. The severity of AA is based on the Camitta criteria, which include severe aplastic anemia (SAA), very severe aplastic anemia (vSAA) and nonsevere aplastic anemia (nSAA) (4–6). The median age at diagnosis among children and adolescents is 8–9 years (7).

Acquired AA is an immune-mediated bone marrow failure disorder characterized by severe pancytopenia and marrow hypoplasia, with high risks of life-threatening hemorrhage and severe infection without standardized intervention (8). The theraputic strategies for pediatric SAA/vSAA currently consist of allogeneic hematopoietic stem cell transplantation (HSCT) and immunosuppressive therapy (IST), alongside long-term supportive care (SC) (9). For newly diagnosed pediatric SAA patients with a human leukocyte antigen (HLA)-matched sibling donor (MSD), upfront transplantation remains the gold-standard curative approach; however, less than half of children can access an ideal fully MSD at initial diagnosis, restricting universal application of this modality (10). For patients lacking MSD, standard IST combining antithymocyte globulin (ATG) and cyclosporine A (CSA) serves as the conventional first-line regimen, with thrombopoietin receptor agonists (TPO-RAs) such as eltrombopag (EPAG) increasingly incorporated to accelerate hematopoietic recovery and elevate complete response rates (10). Haploidentical HSCT has rapidly matured over the past decade as an alternative transplant modality, resolving donor shortage barriers, yet it is complicated by substantial risks of graft-vs.-host disease (GVHD), graft rejection and prolonged post-transplant immune reconstitution defects (9). For nSAA, current guidelines have not reached a unified therapeutic consensus. Some researchers recommend regular observation only, while others favor alternative interventions including IST, androgen or TPO-RAs (11). Despite remarkable improvements in short-term response and overall survival (OS) across all therapeutic branches, prominent clinical dilemmas persist in real-world pediatric practice. Approximately one-third of children receiving standard IST fail to achieve durable complete remission, accompanied by recurrent relapse, persistent transfusion dependence and cumulative risks of clonal evolution during long-term follow-up (9). Substantial heterogeneity exists in treatment efficacy and adverse event profiles across distinct pediatric age subgroups, while most published clinical evidence relies on short-term cohort observations with limited decade-scale longitudinal data to compare long-term safety, late complications and predictive biomarkers between IST and different transplant platforms (9).

Current guidelines lack unified stratified analytical evidence regarding long-term outcomes of multi-line therapeutic sequences in pediatric AA populations, highlighting an urgent unmet clinical demand for comprehensive long-term real-world cohort analyses to refine individualized pediatric AA management.

This study summarizes a 10-year single-center clinical experience in the management of children with acquired AA, including patients with IST-refractory AA and nSAA. We predefined clear primary and secondary endpoints to systematically analyze long-term therapeutic efficacy, adverse event spectrum, subgroup-specific treatment responses, OS and event-free survival (EFS).

## Methods

This was an ambispective cohort study. Baseline clinical data were retrospectively extracted from electronic medical records, including anonymized study ID, gender, age at onset, potential precipitating factors, clinical manifestations, physical signs, comorbidities, baseline complete blood count (CBC), date of diagnosis, and disease severity. Information regarding the initiation time and treatment modalities was collected. Prespecified evaluation time points were set at 1, 3, 6, 12, and 24 months after treatment initiation. CBC results recorded in hospital laboratory systems at corresponding time points were retrieved preferentially. Telephone follow-up was conducted to supplement clinical information (e.g., CBC, medication adherence, relapse, adverse events and survival status) for patients without available in-hospital laboratory data. Collected follow-up outcomes included treatment modifications, hematologic responses of varying degrees, severe treatment-related adverse events, disease progression/clonal evolution, all-cause mortality, and loss to follow-up. Patients who could not be successfully contacted and lacked any outpatient or inpatient records by the cutoff date of final follow-up were defined as loss to follow-up. Follow-up data were collected until the data cutoff date of April 28, 2026. Patients were censored at the time of death, loss to follow-up, or April 28, 2026, whichever occurred first. Treatment-related adverse events were graded according to the Common Terminology Criteria for Adverse Events (CTCAE), version 5.0. Adverse events of Grade ≥3 were defined as severe adverse events. Baseline data were extracted independently by two senior hematologists; discrepancies were resolved by re-reviewing source medical records. This study was approved by the Institutional Review Board of Shanghai Children's Hospital. Written informed consent was obtained from the guardians of all enrolled patients. In addition, per the committee's requirements, written assent was obtained from children aged 8 years or older, while verbal assent was acquired from children younger than 8 years old.

## Patient eligibility

Inclusion criteria:

1. Age younger than 18 years; diagnosed with childhood AA and classified according to the Camitta criteria;
2. Newly diagnosed from January 2015 to February 2024 and received standardized regular treatment in our hospital;
3. Complete clinical medical records, with available baseline data including CBC, bone marrow aspiration results and immune function indicators;
4. Prognosis and efficacy data could be collected via outpatient reexamination or telephone follow-up.

Exclusion criteria:

1. Children with congenital/inherited bone marrow failure syndromes, secondary AA or other hematopoietic failure diseases;
2. Complicated with malignant tumors, severe autoimmune diseases, severe hepatorenal insufficiency and other critical comorbidities;
3. Receiving targeted treatments such as IST or HSCT at other hospitals before admission;
4. Patients with severely missing clinical data or loss to follow-up, who were unavailable for efficacy evaluation and survival outcome analysis.

## Deﬁnitions

### Treatment definitions

Initial treatment: The therapeutic regimen administered immediately after diagnosis of AA, including Standard IST combined with/without TPO-RA, CSA combined with/without TPO-RA, HSCT and SC (including red blood cell and platelet transfusions, iron chelation, infection prophylaxis, and symptomatic management).

Ultimate therapy is defined as the highest-tier treatment for AA administered over the entire disease course, with the priority hierarchy ranked as allogeneic HSCT > standard IST > cyclosporine A > SC.

Response to treatment was defined as the achievement of partial or complete response (12): complete response (CR) for AA was deﬁned as transfusion independence, hemoglobin (Hb) ≥ 100 g/L, absolute neutrophil count (ANC) ≥ 1.5 × 10^3^/μL and platelet (PLT) ≥ 100 × 10^3^/μL; partial response (PR) for SAA/vSAA patients was deﬁned as transfusion independence and Hb ≥ 80 g/L, ANC > 0.5 × 10^3^/μL and PLT > 20 × 10^3^/μL (with at least two out of the three parameters being demonstrated) but the lack of attainment of CR; PR for nSAA patients was deﬁned as transfusion independence (if previously dependent) or doubling or normalization of at least one cell line or increase of baseline (Hb concentration of >30 g/L [if initially <60]; ANC of >0.5 × 10^3^/μL [if initially <0.5]; PLT of >20 × 10^3^/μL [if initially <20]) but a lack of attainment of CR. All of the response had to be conﬁrmed by at least 2 blood counts obtained at least 4 weeks apart. None response (NR) was defined as worse blood counts or without meeting criteria of PR.

OS was defined as the time from the initiation of treatment to all-cause death. Event-free survival was defined as the interval from the first day of initial treatment to the first occurrence of event (including treatment failure at 6 months, disease relapse, clonal hematological disease progression, or all-cause mortality) (13). For patients receiving HSCT, transplant-specific variables were collected and defined as follows:

Donor type: categorized as MSD, matched unrelated donors (MUD), and haploidentical related donor (HID).

Acute GVHD (aGVHD): graded 0–IV within 100 days post-transplant; chronic GVHD (cGVHD) diagnosed after day 100.

Graft failure: Primary graft failure was defined as sustained pancytopenia without hematopoietic engraftment by day 28; secondary graft failure referred to recurrent cytopenia after initial successful engraftment.

Severe transplant-related toxicity: Severe organ toxicities (hepatic, renal, pulmonary, mucosal) grade ≥3 per CTCAE 5.0 occurring within 1 year after HSCT.

## Statistical methods

All statistical analyses were performed using GraphPad Prism 9.5 (GraphPad Software, La Jolla, CA, USA). Survival curves were plotted by the Kaplan–Meier method and compared with the log-rank test. Differences in the subgroups were evaluated with the chi-square test for nominal values and the Mann–Whitney *U*-test and Fisher's exact test for continuous variables. A *p* value of <0.05 (two-tailed) was considered to be statistically significant.

## Results

### Patient characteristics

A total of 70 children with AA were enrolled in this study, including 19 patients with nSAA, 30 with SAA, and 21 with vSAA. The median age of participants was 7.2 (0.9, 14.0) years, and the male-to-female ratio was 0.56:0.44. The median follow-up duration was 60.7 (0.4, 136.5) months. Baseline CBC parameters across the three groups are summarized in Table 1.

**Table 1:** Characteristics of patients with AA.

Characteristic	*N* (%)/median (range)			*p*
	nSAA (*n* = 19)	SAA (*n* = 30)	vSAA (*n* = 21)	
Age (years)	6.1 (1.6,11.7)	7.1 (1.0,12.4)	5.0 (0.9,14.0)	0.84
Sex (male)	6 (31.6)	18 (60.0)	15 (71.4)	0.036
CBC parameters				
WBC (10^3^/μL)	3.79 (2.19,5.32)	1.43 (0.9,2.7)	1.79 (1.07,3.68)	<0.001
Hb (g/L)	100.5 (66,127)	75 (32,108)	74.5 (43,108)	0.003
ANC (10^3^/μL)	1.08 (0.41,1.59)	0.39 (0.2,1.05)	0.12 (0.01,0.19)	<0.001
PLT (10^3^/μL)	34.5 (9,72)	8 (1,16)	3.5 (2,17)	<0.001

*p* < 0.05 was considered statistically significant.

AA, aplastic anemia; SAA, severe aplastic anemia; vSAA, very severe aplastic anemia; nSAA, non-severe aplastic anemia; CBC, complete blood count; WBC, white blood cell; Hb, hemoglobin; ANC, absolute neutrophil count; PLT, platelet.

### Therapeutic regimen

The therapeutic regimen implemented was based on the Guidelines for the Diagnosis and Management of AA (12, 14). In this study, real-world initial treatment regimens included CSA, rabbit anti-thymocyte globulin (rATG) combined with CSA (standard IST), TPO-RA (e.g., EPAG, Hetrombopag, Avatrombopag) combined IST, HSCT and SC. CSA was orally administered at a starting dose of 2.5 mg/kg every 12 h, with a goal trough level of 200–400 ng/mL, adjusted based on the response and toxicity. The dose of rATG was 3.5–5 mg/kg/day for 5 days. Graft sources for HSCT were peripheral blood stem cells obtained from MSD, MUD or HID. The initial treatment regimen distribution for each group is summarized in Table 2.

**Table 2:** Treatment distribution of patients with AA.

Treatment	*N* (%)/median (range)			*p*
	nSAA (*n* = 19)	SAA (*n* = 30)	vSAA (*n* = 21)	
Initial therapy				0.105
SC	5 (26.3)	1 (3.3)	1 (4.8)	
CSA	12 (63.2)	14 (46.7)	12 (57.1)	
^s^IST	0 (0.0)	3 (10.0)	2 (9.5)	
CSA + TPO-RA	1 (5.3)	2 (6.7)	0 (0.0)	
^s^IST + TPO-RA	1 (5.3)	5 (16.7)	2 (9.5)	
HSCT	0 (0.0)	5 (16.7)	4 (19.0)	
Ultimate therapy				0.001
SC	3 (15.8)	1 (3.3)	2 (9.5)	
CSA	8 (42.1)	1 (3.3)	3 (14.3)	
^s^IST	0 (0.0)	1 (3.3)	4 (19.0)	
CSA + TPO-RA	3 (15.8)	3 (10.0)	0 (0.0)	
^s^IST + TPO-RA	1 (5.3)	5 (16.7)	3 (14.3)	
HSCT	4(21.1)	19(63.3)	9(42.9)	

*p* < 0.05 was considered statistically significant.

AA, aplastic anemia; SAA, severe aplastic anemia; vSAA, very severe aplastic anemia; nSAA, non-severe aplastic anemia; SC, supportive care; CSA, cyclosporine A; ^s^IST, standard immunosuppressive therapy; TPO-RA, thrombopoietin receptor agonist; HSCT, hematopoietic stem cell transplantation.

Among all 70 patients, HSCT (32/70, 45.7%), represented the most frequently administered ultimate therapy, which was followed by CSA monotherapy (12/70, 17.1%), standard IST combined with TPO-RA (9/70, 12.9%), CSA plus TPO-RA (6/70, 8.6%), SC (6/70, 8.6%), and standard IST alone (5/70, 7.1%). Stratified analysis demonstrated that HSCT constituted the predominant treatment strategy in patients with SAA/vSAA (28/51, 54.9%), followed by standard IST plus TPO-RA (8/51, 15.7%). In contrast, CSA monotherapy was the leading treatment for patients with nSAA, accounting for 42.1% (8/19), while the proportion of HSCT application was only 21.1% (4/19), mainly administered due to disease progression. Pearson's chi-square test revealed a significant difference in the distribution of ultimate therapies between the SAA/vSAA and nSAA groups (Fisher's exact test, *p* = 0.001). These findings indicate a distinct divergence in clinical treatment selection for children with AA across different disease severity subgroups (Table 2).

We summarized transplant-specific clinical characteristics for all patients undergoing HSCT (Table 3). The table presents the breakdown of donor types, cumulative incidences of acute and chronic GVHD, incidence of graft failure, and frequencies of severe transplant-related toxicities.

**Table 3:** Transplant-specific characteristics of patients receiving HSCT.

Variable	*N* (%)
Donor type	
MSD	8 (25.0)
MUD	10 (31.3)
HID	14 (43.7)
Cumulative incidence of grade II–IV aGVHD (Day 100)	7 (21.8)
Cumulative incidence of cGVHD (2 years)	2 (6.3)
Overall graft failure rate	1 (3.1)
Rate of grade ≥3 transplant toxicity	4 (12.5)

MSD, human leukocyte antigen (HLA) matched sibling donor; MUD, HLA matched unrelated donors; HID, HLA-haploidentical related donor; aGVHD, acute graft-versus-host disease; cGVHD, chronic graft-versus-host disease.

### Treatment response evaluation

**Figure 1:**
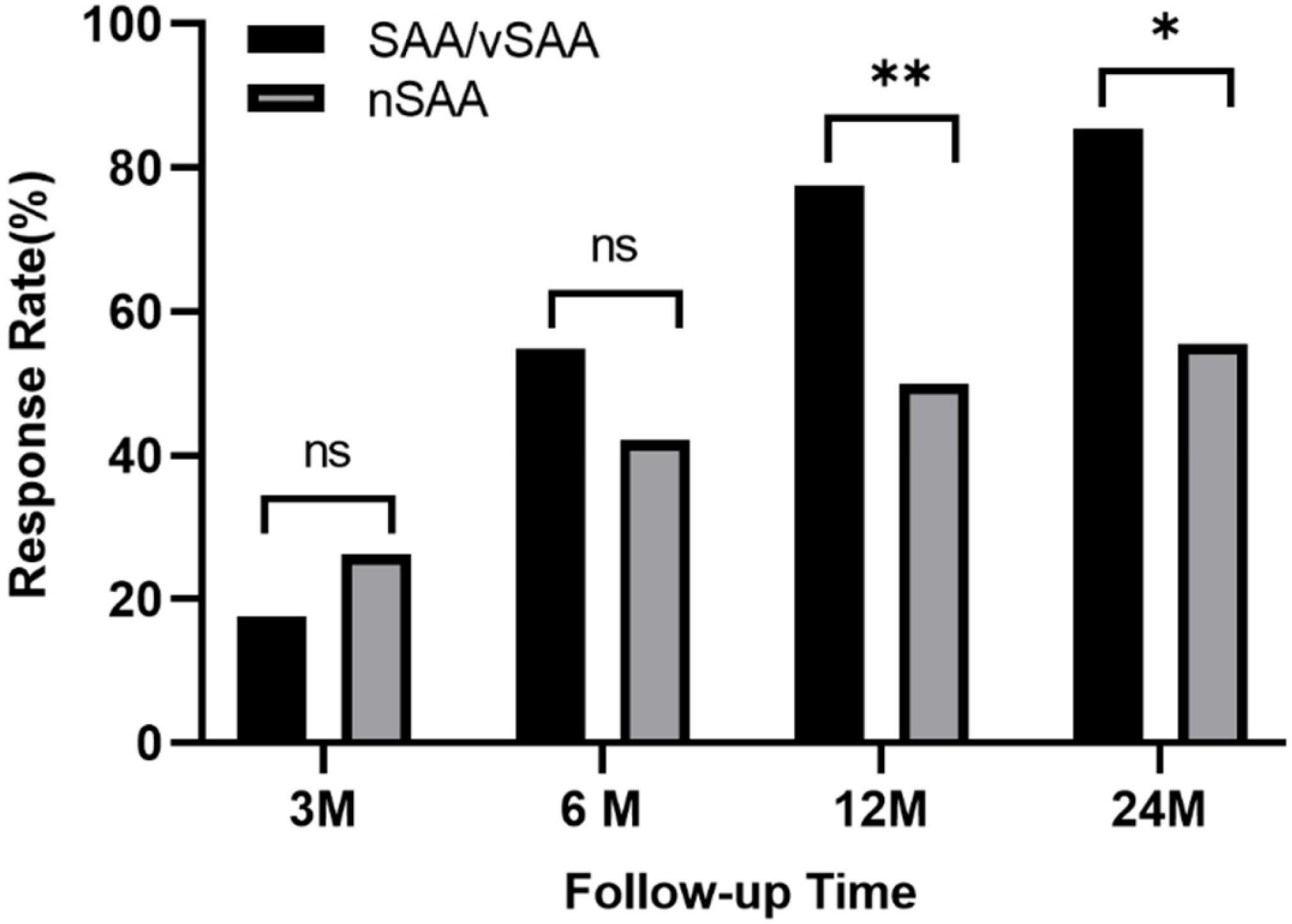
Treatment response rates of patients with AA at 3-month, 6-month, 12-month and 24-month after treatment initiation. Treatment response rates increased over time in both groups. No significant differences were found between the SAA/vSAA group and nSAA group at 3 months (*p* = 0.870) and 6 months (*p* = 0.341). At the 12-month and 24-month evaluation, the SAA/vSAA group showed a significantly higher RR than the nSAA group (*p* = 0.007, *p* = 0.019). Chi-square test was used for ratio comparison analysis. *p* < 0.05 was considered statistically significant.

To evaluate the therapeutic efficacy in patients with AA, we further analyze the treatment response at 3 months, 6 months, 12 months and 24 months after treatment initiation (Figure 1).

In patients with SAA/vSAA (*n* = 51 at baseline), the response rate (RR) was 17.7% at 3 months, consisting of 15.7% PR and 2.0% CR. At 6 months, the RR increased to 54.9%, with 33.3% PR and 21.6% CR. At the 12-month follow-up, 49 patients remained evaluable; the RR reached 77.6%, including 24.5% PR and 53.1% CR. Two patients were lost to response assessment at this time point. At the 24-month evaluation (48 evaluable patients), the RR further rose to 85.5%, comprising 18.8% PR and 66.7% CR.

For patients with nSAA (*n* = 19 at baseline), the RR was 26.3% at 3 months; all responders achieved PR, and no CR was observed. At 6 months, the RR was 42.1%, with PR and CR rates of 36.8% and 5.3%, respectively. At 12 months, 18 patients were evaluable, and the RR was 50.0% (38.9% PR, 11.1% CR); one patient was unavailable for efficacy assessment. At the 24-month follow-up, the RR was 55.6% (38.9% PR, 16.7% CR). During the 2-year observation period, three patients (16.7%) with nSAA progressed to SAA.

Intergroup comparisons revealed statistically significant differences in RR between the SAA/vSAA and nSAA cohorts at 12 months (*χ*^2^ = 9.853, *p* = 0.007) and 24 months (*p* = 0.019), with higher response rates consistently observed in patients with SAA/vSAA.

### Survival analysis

The Kaplan–Meier method was used to plot survival curves, and the Log-rank test was applied for intergroup comparison.

The 2-year OS rate of all patients was 97.1% (SE = 0.02, 95%CI: 0.9318–1.0000).

Stratified analysis by AA subtypes showed that the 2-year OS rate was comparable between the SAA/vSAA group and the nSAA group (96.1% [SE = 0.027, 95% CI: 0.9081–1.0000] vs. 100%).

**Figure 2:**
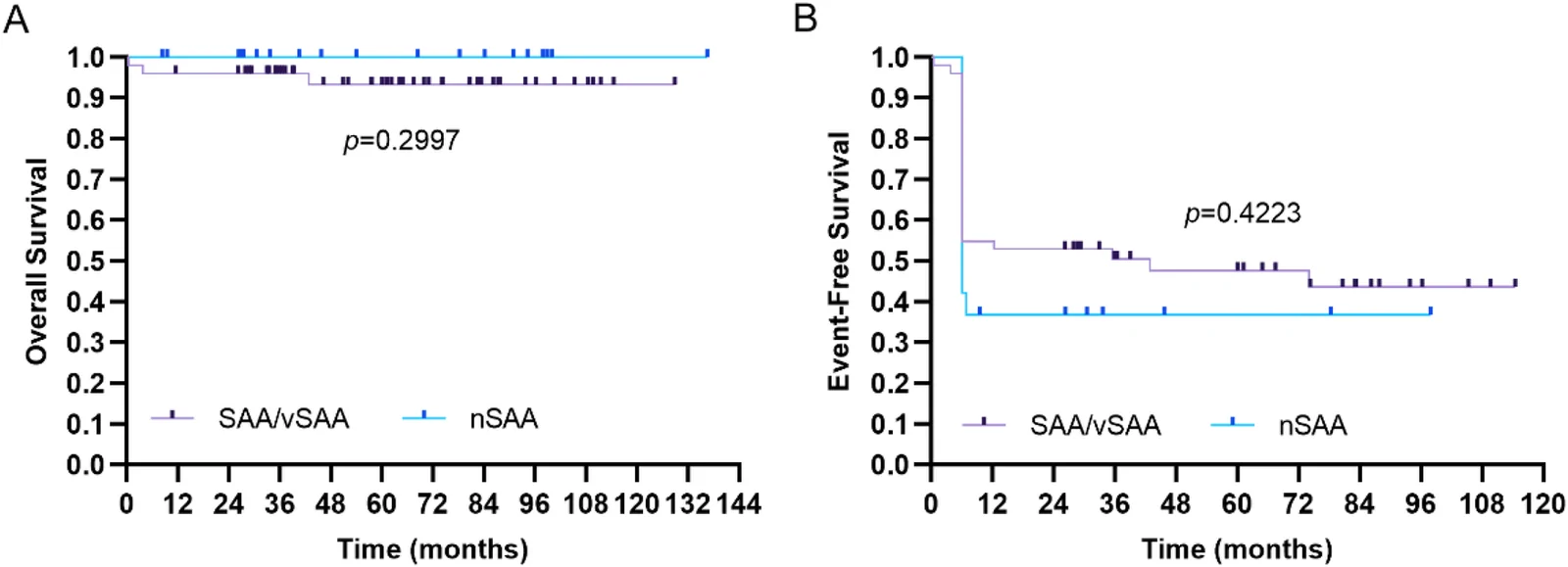
Kaplan–Meier analysis of OS and EFS in patients with AA. **(A)** No deaths occurred in the nSAA cohort (100% OS), while the SAA/vSAA group had an estimated 2-year OS of 96.1%. The difference was not statistically significant (Log-rank test, *p* = 0.2997). **(B)** The SAA/vSAA group showed a numerically higher estimated long-term EFS rate compared with the nSAA group, but the difference was not statistically significant by the log-rank test (*p* > 0.05).

No significant difference was observed in OS curves between the two groups (*p* = 0.2997). These findings indicated that pediatric patients with AA presented a favorable OS outcome (Figure 2A).

The 2-year EFS rate of all patients was 48.5% (SE = 0.06, 95% CI: 0.3674–0.6026). The 2-year EFS rate was 52.9% (SE = 0.07, 95% CI: 0.3918–0.6662) in the SAA/vSAA group and 36.8% (SE = 0.111, 95% CI: 0.1504–0.5856) in the nSAA group, with no significant difference (*p* = 0.4223) (Figure 2B). Quantitative proportions and absolute numbers for each group of adverse event were presented in Table 4.

**Table 4:** Breakdown of the events driving poor EFS.

Group	*N* (%)				
	Treatment failure^#^	Disease relapse	Clonal evolution^*^	All-cause death	Total events
Overall cohort (*n* = 70)	30 (42.9)	5 (7.1)	0 (0.0)	4 (5.7)	39 (55.7)
nSAA (*n* = 19)	11 (57.9)	1 (5.3)	0 (0.0)	0 (0.0)	12 (63.2)
SAA/vSAA (*n* = 51)	19 (37.3)	4 (7.8)	0 (0.0)	4 (7.8)	27 (52.9)
Initial definitive treatment					
IST (*n* = 6)	3 (50.0)	0 (0.0)	0 (0.0)	0 (0.0)	3 (50.0)
HSCT (*n* = 8)	0 (0.0)	0 (0.0)	0 (0.0)	0 (0.0)	0 (0.0)

# Treatment failure at 6 month;

* clonal hematological disease progression.

SAA, severe aplastic anemia; vSAA, very severe aplastic anemia; nSAA, non-severe aplastic anemia; IST, standard immunosuppressive therapy; HSCT, hematopoietic stem cell transplantation.

### Effects of different treatment regimens on treatment response and survival

First, we analyzed the correlations between different initial therapy regimens and treatment responses at 3 and 6 months in patients with nSAA. No significant effect of initial therapy selection on short-term therapeutic responses was observed (*p* > 0.05). Similarly, no significant difference in 2-year treatment outcomes was identified among patients with nSAA receiving different ultimate therapy regimens (*p* > 0.05).

**Figure 3:**
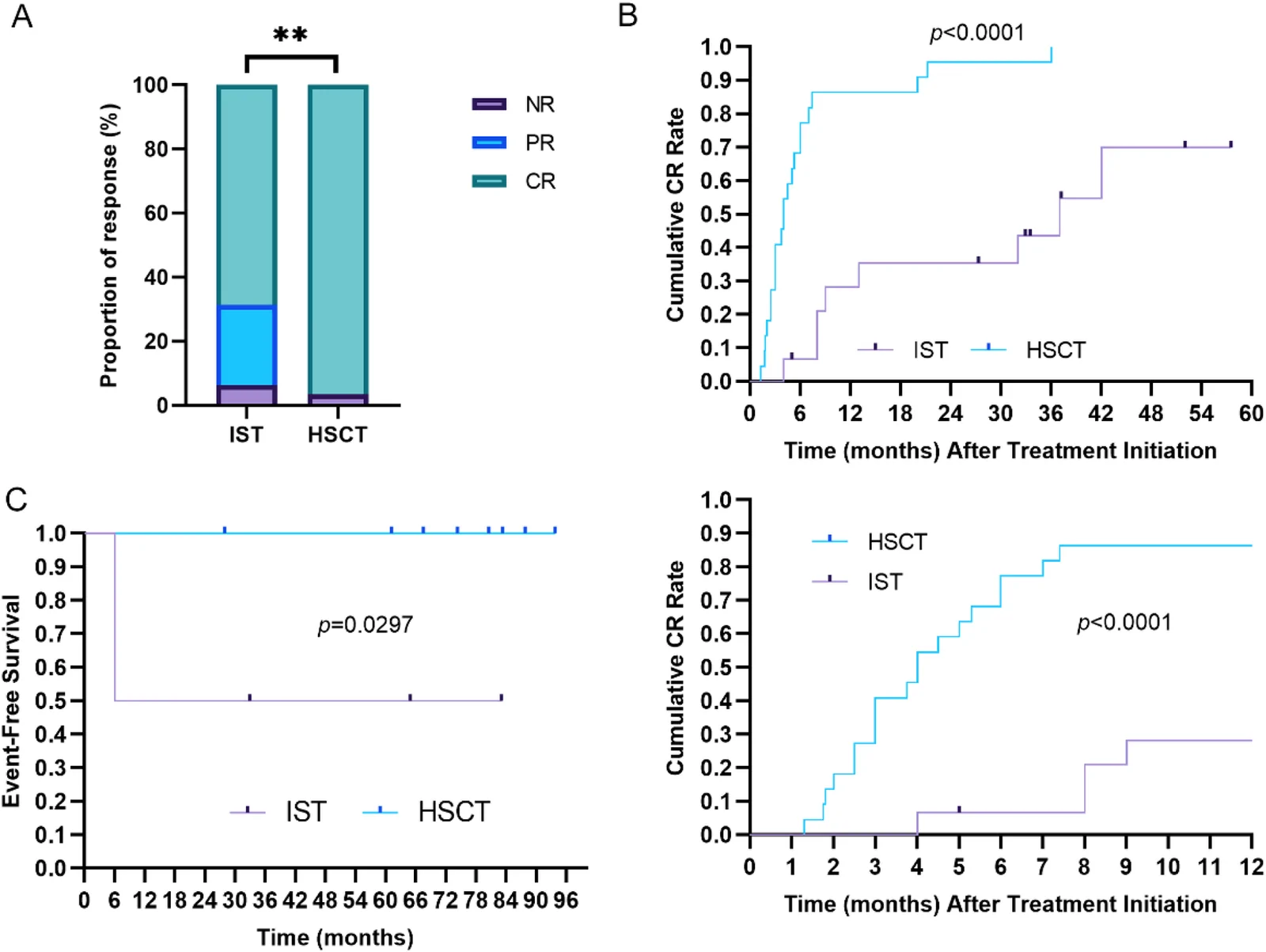
Effects of different therapeutic regimens on treatment response and survival. **(A)** The constituent ratios of NR, PR and CR remission stratification were significantly different between the IST group and the HSCT group (*p* = 0.007). **(B)** Kinetics of CR in patients with SAA/vSAA treated with HSCT versus IST. Kaplan–Meier curves showing the cumulative proportion of patients achieving CR over time. The HSCT group exhibited a significantly higher and faster cumulative CR rate compared with the IST group (log-rank test, *p* < 0.0001). **(C)** EFS according to treatment strategy. Kaplan–Meier curves comparing EFS between patients receiving HSCT versus IST as first-line therapy. The HSCT group demonstrated a significantly higher EFS rate compared with the IST group (log-rank test, *p* = 0.0297).

We analyzed the disease treatment response at the final follow-up in patients with SAA/vSAA who received IST or HSCT as the definitive therapy regimen. The overall RR was 95.5%, with a CR rate of 86.4% and a PR rate of 9.1%. In the IST group, 4 patients (25.0%) achieved PR and 11 patients (68.8%) achieved CR, while 1 patient had NR. In the HSCT group, 27 patients (96.4%) achieved CR and 1 patient (3.6%) had NR; no patients attained PR in this group. The distribution of disease remission status appeared to differ notably between the two groups (Fisher's exact test, *p* = 0.007). The HSCT group had a higher proportion of CR and exhibited superior overall treatment response compared with the IST group (Figure 3A).

Time to CR was analyzed using the Kaplan–Meier method, where the endpoint was defined as the achievement of CR. Patients who did not achieve CR by the end of follow-up, were lost to follow-up, or died prior to CR were considered censored observations. The cumulative CR rate was significantly higher in the HSCT group than in the IST group throughout the 60-month follow-up period (log-rank test, *p* < 0.0001). The HSCT group began achieving CR as early as 1 month+ and achieved a rapid increase in CR rate within the first 12 months. In contrast, the IST group showed a slower and lower rate of CR attainment, with a gradual increase starting after 3 months (Figure 3B).

No death occurred in SAA/vSAA patients who received initial standard IST (*n* = 6) or HSCT (*n* = 8). Therefore, we further analyzed the effect of the two treatment regimens on EFS. Breakdown of the events driving poor EFS were presented in Table 4. Patients treated with HSCT exhibited a trend toward improved 2-year EFS compared to patients receiving IST (100% vs. 50%; Log-rank test, *p* = 0.0297) (Figure 3C).

### Clinical efficacy of EPAG in children with IST-resistant SAA/vSAA

**Figure 4:**
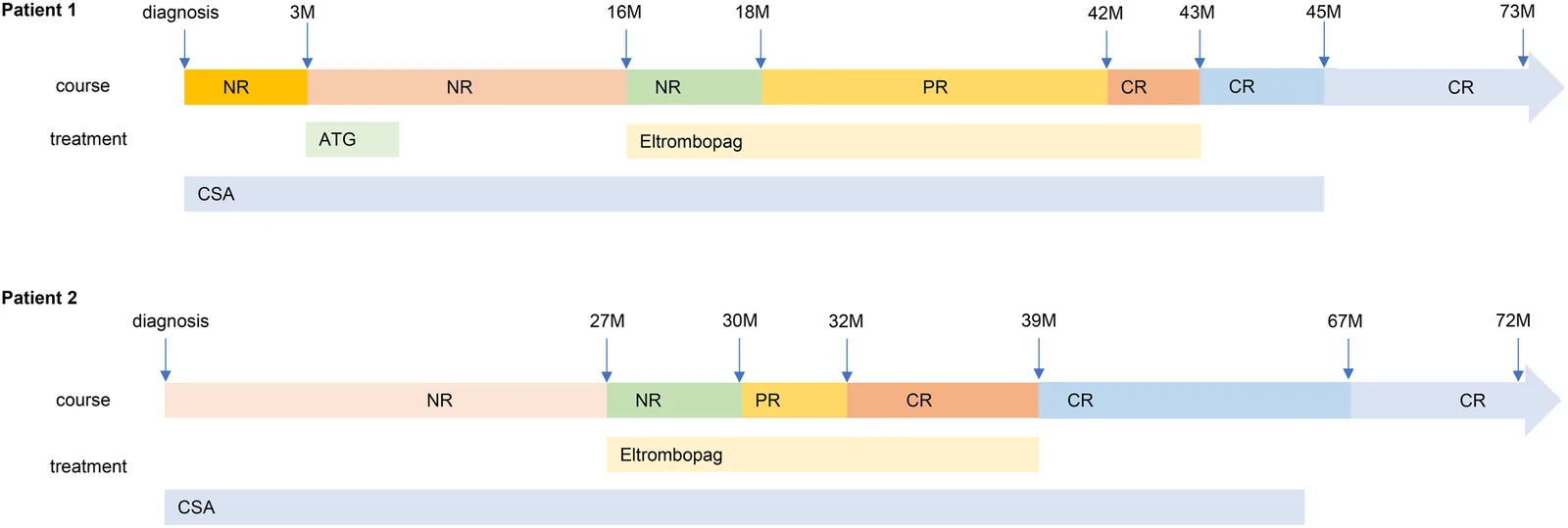
Clinical courses and treatment responses of two children with IST-resistant SAA/vSAA who received eltrombopag as salvage therapy. Patient 1 had persistent NR after initial IST at 3 and 16 months. Eltrombopag was added to CSA at 16 months. PR was achieved at 18 months and CR at 42 months, which was sustained through 73 months of follow-up. Patient 2 exhibited NR to first-line CSA-based IST. After eltrombopag was added at 27 months, PR was achieved at 30 months and CR at 32 months, which persisted through 72 months of follow-up. M, months.

In this study, We observed a trend toward favorable outcomes associated with EPAG in pediatric patients, who did not respond to IST. Specifically, a 4-year-old girl with vSAA received CSA followed b**y** standard IST, but she still demonstrated NR to the IST over the course of 16 months. Subsequently, EPAG was added to the patient's ongoing CSA regimen. The patient achieved PR and CR after 2 months and 26 months, respectively. Moreover, the treatment periods for CSA and EPAG were 45 months and 27 months, respectively (Figure 4 Patient 1). The second patient was a 3-year-old boy diagnosed with SAA, who received CSA as initial and long-term treatment. After 27 months, the patient still did not respond to CSA; thus, he received EPAG in conjunction with CSA. After the combination regimen was initiated, the boy achieved PR and CR at 3 months and 5 months, respectively (Figure 4 Patient 2). In total, the treatment period were 67 months and 12 months for CSA and EPAG, respectively. Neither patient discontinued EPAG due to liver toxicity or other side effects. Furthermore, no evidence of relapse or clonal evolution was observed in both two patients.

## Discussion

Pediatric acquired AA is a rare, potentially fatal disease that results from immune-mediated destruction of hematopoietic stem cells and progenitor cells (15). Notably, SAA/vSAA present rapid disease progression with high risks of bleeding and infection, and harbor a poor long-term prognosis (16, 17). Differences in efficacy among various treatment regimens, prognostic influencing factors, and optimized therapeutic strategies for IST-resistant patients have long been research hotspots in the clinical field of AA. In this study, we retrospectively analyzed the clinical data of children with AA, summarized their baseline clinical characteristics, compared the short-term efficacy and long-term survival outcomes of different treatment regimens, and explored the clinical value of EPAG in pediatric patients with IST-resistant SAA/vSAA.

This study demonstrated that the therapeutic regimens differed significantly among children with different disease severity. Patients with nSAA were mainly treated with oral CSA alone or supportive care, while children with SAA/vSAA primarily received HSCT, standard IST, or combined therapy with TPO-RAs. Limited by the relatively small sample size of nSAA patients in this study, no significant difference in treatment response was observed among different regimens for nSAA. In the present cohort, among the five nSAA patients who initially received SC, only two achieved CR; 16.7% of total nSAA children progressed to SAA, indicating that nSAA is not always associated with a benign clinical course. There is no uniform position in literature on the treatment of nSAA. Some experts suggest in nSAA with bleeding, frequent transfusion requirements and high risk of infection, IST or HSCT is indicated as in SAA (11).

IST is currently the first-line treatment for SAA. The combination of CSA and ATG can inhibit abnormally activated T lymphocytes and relieve immune-mediated hematopoietic suppression in the bone marrow. Nevertheless, clinical data indicate that 20%–30% of patients with SAA have a poor response to IST, characterized by delayed hematological recovery and persistent transfusion dependence, eventually progressing to IST resistance (17). For children with IST-resistant SAA/vSAA, conventional immunosuppressive regimens hardly achieve satisfactory hematopoietic reconstitution. Repeated long-term blood transfusions may lead to multiple complications such as iron overload and recurrent infection, which seriously impair the quality of life of pediatric patients. Therefore, optimized therapeutic strategies are urgently required for resistant cases.

TPO-RAs, especially for adult patients, have been widely applied in newly diagnosed and refractory/relapsed AA in recent years (2, 18–20). The present study described that the combination of EPAG improve the hematological response rate in two children with IST-resistant SAA/vSAA, which is consistent with previous pediatric studies (8). However, the limited sample size was insufficient to draw any definitive inference; and large-scale prospective cohorts are required to validate eltrombopag's clinical value in pediatric AA. EPAG not only promotes megakaryocyte proliferation and differentiation but also acts on hematopoietic stem and progenitor cells to improve the bone marrow hematopoietic microenvironment. Additionally, it exhibits mild immunomodulatory effects that help correct immune tolerance disorders, which explains the improved efficacy after EPAG administration in resistant patients (19, 21). In this study, no hepatic and renal impairment, as well as clonal evolution were observed in both patients.

Survival analysis revealed that the OS rate of children with AA in this cohort was favorable, whereas the EFS rate was unsatisfactory, with a 2-year EFS of approximately 50%. Notably, children with nSAA did not demonstrate superior EFS outcomes. Treatment failure and disease relapse constituted the predominant clinical events associated with unfavorable EFS outcomes in this patient population. Among patients with SAA/vSAA, individuals treated with IST as definitive therapy appeared to experience higher risks of treatment failure and disease recurrence, alongside shorter EFS. In contrast, HSCT may facilitate rapid hematopoietic reconstitution and attainment of CR, with potential improvements in EFS consistent with trends observed in prior studies (13, 22, 23). We systematically outline several alternative confounding factors that may account for the favorable survival outcomes seen among HSCT recipients: imbalanced baseline clinical profiles (patients selected for HSCT were younger at diagnosis and had shorter periods of transfusion dependence), selection bias (only patients free from severe end-organ comorbidities qualified for allogeneic HSCT), and refined contemporary transplant supportive care protocols. Standardized infection prophylaxis, GVHD prophylaxis, and transfusion support independently lower transplant-related mortality, independent of the intrinsic curative capacity of HSCT itself.

HSCT is the preferred and curative treatment option for pediatric patients with acquired AA when considering that late clonal disorders such as Myelodysplastic Syndrome and Acute Myeloid Leukemia occur in up to 15% of patients who are initially treated with IST (24). However, the transplantation rate was low in our study. Multiple factors jointly led to the low HSCT proportion. First, 27% of the patients were diagnosed with nSAA, for which HSCT was not recommended as the first-line therapeutic strategy in domestic and international clinical guidelines for AA. Second, the majority of parents refused HSCT due to worries about GVHD and transplant-related complications. Third, matched sibling donors were scarce in China, and unrelated donor searching was not routinely performed in the early phase of this cohort. Some patients also had active infections precluding immediate transplantation.

This single-center retrospective study carries multiple limitations: limited sample size, non-randomized design, relatively short follow-up, and long-term follow-up data for a subset of patients were collected via telephone interviews, which may introduce potential recall bias. The small sample led to inherently low statistical power, which prevented reliable implementation of multivariable regression, effect size calculation and sensitivity analyses because of high risk of overfitting. Serial response assessments were not standardized under the retrospective design. Variable follow-up intervals and missing time-point data precluded longitudinal statistical modelling. Patients receiving HSCT generally presented with more severe baseline disease and longer transfusion dependence. Such baseline imbalances may overestimate intergroup efficacy differences and introduce confounding by indication. A subset of patients experienced primary IST failure and subsequently switched to salvage therapy or HSCT. This crossover dilutes the genuine efficacy difference between initial treatment groups and generates confounding effects. Furthermore, for patients receiving delayed definitive therapy, the observational window prior to treatment initiation inherently constitutes immortal time. Larger multicenter cohorts integrating genetic and immune biomarkers are needed to explore independent prognostic factors for children with AA.

In conclusion, our data suggest that HSCT may yield more rapid hematologic remission and favorable EFS outcomes among children with SAA/vSAA. For IST-refractory pediatric SAA/vSAA patients lacking appropriate stem cell donors, combination therapy with TPO-RAs may potentially boost hematologic responses and extend EFS. Nevertheless, the present study carries notable limitations including its single-center retrospective design, small sample sizes within several subgroups, and intrinsic selection biases. Large multicenter prospective investigations are therefore warranted to validate our results prior to widespread implementation of these findings in routine clinical practice.

## Data Availability

The original contributions presented in the study are included in the article, further inquiries can be directed to the corresponding authors.
